# Network pharmacology and molecular docking study on the active ingredients of qidengmingmu capsule for the treatment of diabetic retinopathy

**DOI:** 10.1038/s41598-021-86914-8

**Published:** 2021-04-01

**Authors:** Mingxu Zhang, Jiawei Yang, Xiulan Zhao, Ying Zhao, Siquan Zhu

**Affiliations:** 1 Eye School, Chengdu University of Traditional Chinese Medicine, 37 Shi Er Qiao Road, Jinniu District, Chengdu, 610036 China; 2 National Key Laboratory of Human Factors Engineering, China Astronaut Research and Training Center, Lvyuan Road, Haidin District, Beijing, 100089 China; 3 Department of Ophthalmology, Beijing Anzhen Hospital of Capital Medical University, 2 Anzhen Road, Chaoyang District, Beijing, 100020 China

**Keywords:** Eye diseases, Computational biology and bioinformatics

## Abstract

Diabetic retinopathy (DR) is a leading cause of irreversible blindness globally. Qidengmingmu Capsule (QC) is a Chinese patent medicine used to treat DR, but the molecular mechanism of the treatment remains unknown. In this study, we identified and validated potential molecular mechanisms involved in the treatment of DR with QC via network pharmacology and molecular docking methods. The results of Ingredient-DR Target Network showed that 134 common targets and 20 active ingredients of QC were involved. According to the results of enrichment analysis, 2307 biological processes and 40 pathways were related to the treatment effects. Most of these processes and pathways were important for cell survival and were associated with many key factors in DR, such as vascular endothelial growth factor-A (VEGFA), hypoxia-inducible factor-1A (HIF-1Α), and tumor necrosis factor-α (TNFα). Based on the results of the PPI network and KEGG enrichment analyses, we selected AKT1, HIF-1α, VEGFA, TNFα and their corresponding active ingredients for molecular docking. According to the molecular docking results, several key targets of DR (including AKT1, HIF-1α, VEGFA, and TNFα) can form stable bonds with the corresponding active ingredients of QC. In conclusion, through network pharmacology methods, we found that potential biological mechanisms involved in the alleviation of DR by QC are related to multiple biological processes and signaling pathways. The molecular docking results also provide us with sound directions for further experiments.

## Introduction

One of the microvascular complications of diabetic mellitus (DM), diabetic retinopathy (DR), has become the main cause of irreversible visual impairment worldwide and is affecting a growing proportion of DM patients^1^. For diabetic patients with mild or imperceptible ocular symptoms who do not undergo regular fundus examinations, early diagnosis and effective control are difficult in the initial phase of DR. The traditional DR treatment of laser photocoagulation combined with anti-vascular endothelial growth factor (anti-VEGF) administration has seemed to be a considerably effective strategy for nonproliferative diabetic retinopathy (NPDR, diabetic retinopathy without neovascularization) patients^2^. Other treatments for DR include oral calcium dobesilate treatment, insulin administration, weight loss, control of blood pressure and lipids, and even surgery for proliferative diabetic retinopathy (PDR, diabetic retinopathy with neovascularization) patients with retinal detachment^3^. Recently, some new drugs have been used in in vivo or in clinical trials (such as canakinumab and doxycycline)^4^. With regard to molecular biology, neovascularization induced by vascular endothelial growth factor (VEGF) has been proposed to play a leading role in the development of PDR.

Diverse herbs and natural products are used in China as complementary treatments for DM. However, the effects and mechanisms of the ameliorative effects of traditional Chinese medicines (TCMs) on DR remain controversial and unclear, and proper methods are needed to explore and fully explain them in detail. A Chinese patent medicine used to treat DR, Qidengmingmu Capsule (QC), contains 3 herbs: *Erigeron breviscapus*, *Astragalus membranaceus* and Radix Puerariae. *Erigeron breviscapus* has been proven to exert anti-insulin resistance and anti-oxidative stress effects in the treatment of DM^5^. The extract of *Astragalus membranaceus*, Astragalus polysaccharide (APS), also exerts validated therapeutic effects on DM by increasing insulin sensitivity through the AMPK pathway^6^. In a clinical trial performed by Xie, patients with nonproliferative DR who did not meet the indications of retinal photocoagulation or anti-VEGF treatment received QC therapy for 12 weeks. The results showed significant improvements in visual acuity, symptom complaints and retinal lesions. The safety of QC was also verified by the biochemical stability of blood and secretions during the whole treatment period and even 3 months later^7^. TCMs have been developed for thousands of years, and abundant formulae have been created by TCM practitioners to alleviate patients’ suffering. However, it is difficult to modernize TCM through conventional pharmacological and biochemical methods due to the complex ingredients and unclear mechanisms. Network pharmacology, a revolutionary field based on systematic biology and network analysis, provides feasible and reliable ways to explore potential molecular mechanisms beyond the barriers associated with the current one drug-one target-one pathway research mode^8^. Based on the interactions between active herbal ingredients and potential targets of disease, network pharmacological methods can identify the potential mechanisms and key targets from network topological analysis. These methods can also be used to identify synergistic effects of herbal ingredients, to understand combinatorial rules of TCM formulae, and ultimately to help clinical practitioners design herbal formulae rationally^9^. The molecular docking technique plays an important role in drug-target binding prediction and synthetic drug design, enabling prediction of docking patterns and binding affinities (indicating the stability of docking patterns) of drug ligands and protein receptors. To explore and validate the potential mechanism of QC in the treatment of DR, we performed this study based on the methods mentioned above.

## Materials and methods

### Active ingredients of QC and QC-related target screening

Information about the ingredients of QC was obtained from the Traditional Chinese Medicine Systems Pharmacology online platform (https://tcmspw.com/tcmsp.php, TCMSP, Version 2.3) by searching the keywords “Erigeron Breviscapus”, “Astragalus membranaceus” and “Radix Puerariae”^10^. The current formula used by TCM practitioners contains multiple and complicated ingredients, but not all of them have therapeutic effects on disease. Oral bioavailability (OB) represents the ability of a drug to enter the circulation. Druglikeness (DL), a valuable parameter influenced by molecular physical and biochemical properties, represents the similarity between a molecule and known drugs. A desirable OB and DL suggest that a drug candidate has properties that make it suitable for further study^11,12^. The current computationally oriented drug discovery strategy integrates multiple assessments, such as quantitative estimate of druglikeness (QED) and Lipinski's rule-of-five (Ro5, a rule used to predict drug-likeness) assessments^13,14^. In the present study, we selected ingredients with criteria of an OB ≥ 30% and a DL ≥ 0.18 based on similar previous studies^15–17^. Details about the corresponding targets of the active ingredients above were acquired from the TCMSP platform and validated from existing studies. The UniProt database (https://www.uniprot.org, updated on August 12, 2020) was used to obtain gene symbols and related information about the QC targets acquired from TCMSP.

### DR-related target screening

The keyword “Diabetic Retinopathy” was imported to acquire gene symbols of DR-related targets from the GeneCards (Version 5.0) and OMIM (updated on August 31, 2020) databases. We also obtained a BeadChip gene expression dataset (GSE60436) and a platform file (GPL6884) on 3 healthy retina samples and 6 retinal fibrovascular membrane (FVM, pathological tissue of PDR) samples of DR patients from the NCBI-GEO database. Differential gene expression analysis was performed with the Limma packages in R software (version 3.6.0) under the screening condition of an absolute logarithm of the fold change (log FC) value > 0.5. Heatmaps and volcano plots of the differentially expressed genes were created with the Pheatmap package of R software. Finally, we summarized the differentially expressed genes acquired from GEO and the gene symbols acquired from GeneCards and OMIM as the total DR-related targets.

### Active ingredient-DR target and PPI network construction

The QC targets intersecting with DR-related targets were taken as QC-DR common targets. With the assistance of the VennDiagram package of R, we visualized the common targets with a Venn diagram. Cytoscape software (version 3.7.2) was used to construct an active ingredient-DR target network based on the active ingredients of QC and the QC-DR common targets. The different nodes represent DR targets, common targets and QC active ingredient targets, and the nodes are connected by edges (lines), which denote interactions between the nodes. The degree value of a node is denoted by the number of linked edges, which is consistent with the importance of the node. We imported the common targets into the STRING platform (version 11.0) and set the species to *Homo sapiens* and the confidence to 0.950 to obtained the concise protein–protein interaction (PPI) information for further analysis. The PPI network was also visualized with Cytoscape software.

### GO and KEGG enrichment analyses

Using the ClusterProfiler package in R software^18^, we performed Gene Ontology (GO) biological processes and Kyoto Encyclopedia of Genes and Genomes (KEGG) metabolic pathway enrichment analyses on the QC-DR common targets^19–21^. According to the gene ratios, we chose the top 20 biological processes and pathways (P < 0.05) and show them as bubble or column charts that contain the gene counts, gene ratios, adjusted P values and other detailed information. The GO enrichment analysis included 3 categories: the biological process, cellular component and molecular function categories. The results of KEGG enrichment analysis were used to construct a KEGG key pathway network to discover important target proteins involved in the treatment effects of QC. Details about the involved pathways and key targets were downloaded from online platforms to explore the potential mechanisms of the treatment effects on DR.

### Molecular docking validation

Molecular docking, a tool utilized for the prediction and design of new drugs, can simulate intermolecular combinational patterns between drug ligands and target proteins in 3-dimensional (3D) structures to predict possible docking modes and binding affinities. According to the degrees of common targets in the PPI network and their importance in DR etiology, we confirmed key targets and their corresponding active ingredients to perform molecular docking. Molecular 3D structures of active ingredients and key proteins were obtained from TCMSP databases and the RCSB PDB (http://www.rcsb.org/), respectively, and then docking validation was performed after hydrogenation and dehydration through AutoDockTools (version 1.5.6) and AutoDock Vina (version 1.1.2)^22^. Because the functional binding sites of DR-related targets with ingredients of QC remain unknown, all binding sites were limited within the areas of docking pockets (the possible combinational areas of target protein) predicted by DeepSite software (https://www.playmolecule.com)^23^. The molecular docking patterns were visualized with PyMOL (version 2.4).

## Results

### Active ingredient and common target screening

**Figure 1:**
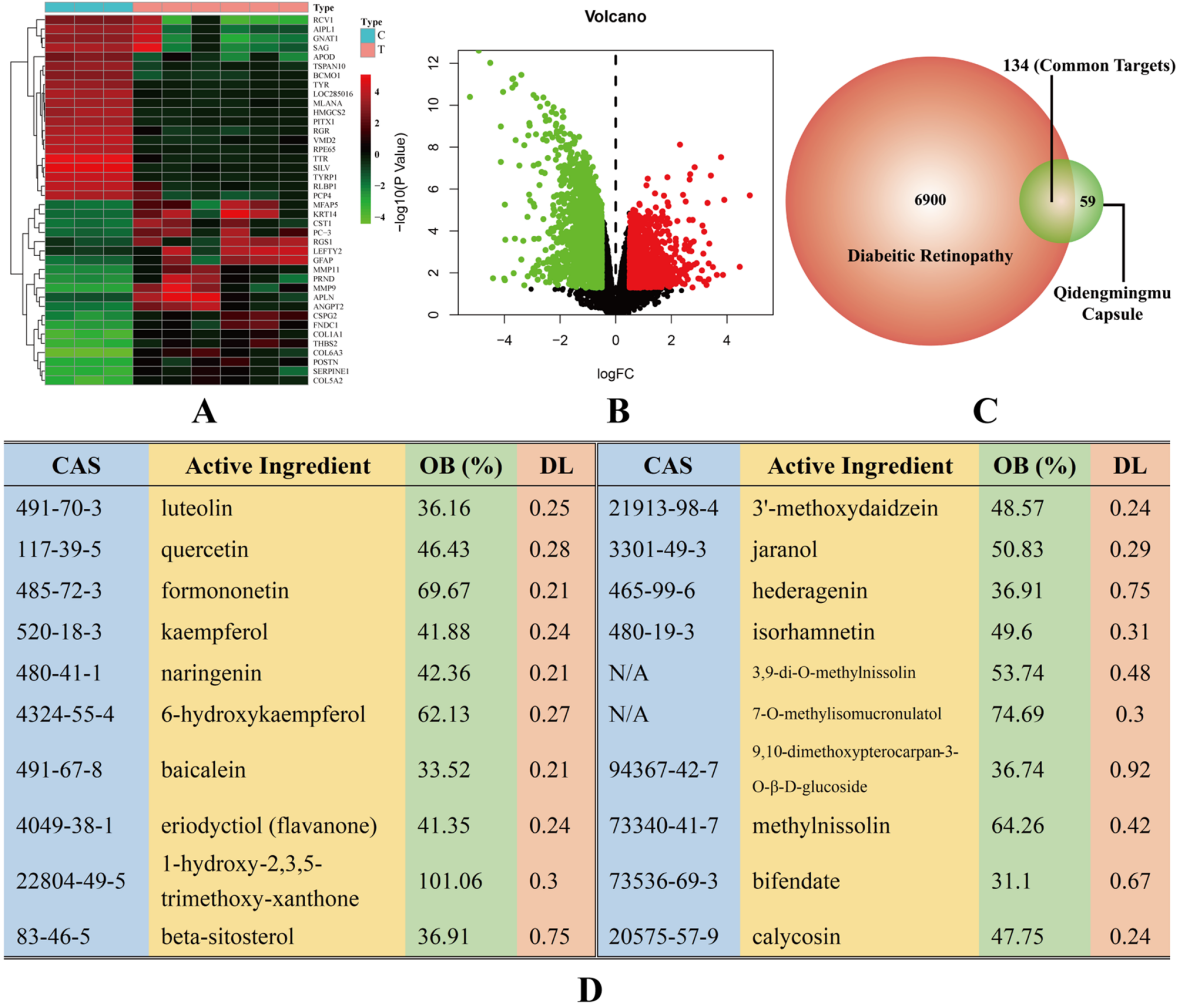
Screening of QC-DR common targets and active ingredients. (**A**) Heatmap of the results from a BeadChip gene expression dataset (GSE60436) in the NCBI-GEO database. Red and green boxes are used to represent upregulation and downregulation, respectively; the control and test groups (Type) are shown in sky blue and carnation. (**B**) Volcano plot of BeadChip gene expression data (GSE60436). Red and green dots are used to represent upregulation and downregulation, respectively; black dots represent nonsignificant changes. (**C**) Venn diagram of QC-DR common targets. (**D**) Details about the active ingredients of QC involved in the treatment of DR.

We obtained 28 ingredients and 193 QC-related targets that satisfied the criteria of an OB ≥ 30% and a DL ≥ 0.18 by searching the keywords “Erigeron Breviscapus”, “Astragalus membranaceus” and “Radix Puerariae” in the TCMSP database. We also obtained 3497, 213 and 4150 DR-related targets from the GeneCards, OMIM and NCBI-GEO databases, respectively. After removal of repetitive gene symbols, we acquired 7034 DR-related targets from the databases above. We generated a heatmap (Fig. 1A) and volcano plot (Fig. 1B) of differentially expressed genes from a BeadChip gene expression dataset (GSE60436) from the NCBI-GEO database. After the DR-related targets and QC-related targets were intersected, we acquired 134 QC-DR common targets (shown in Fig. 1C). By tracing the common targets, we confirmed that 20 active ingredients of QC are involved in the treatment of DR. Details about these active ingredients (chemical name, CAS number, oral bioavailability and druglikeness) are listed in Fig. 1D. The highest OB% was 101.06%, belonging to 1-hydroxy-2,3,5-trimethoxy-xanthone, and the highest DL was 0.75, belonging to beta-sitosterol and hederagenin. The CAS numbers of 3,9-di-O-methylnissolin and 7-O-methylisomucronulatol are not available.

### Ingredient-DR target and PPI network analysis

**Figure 2:**
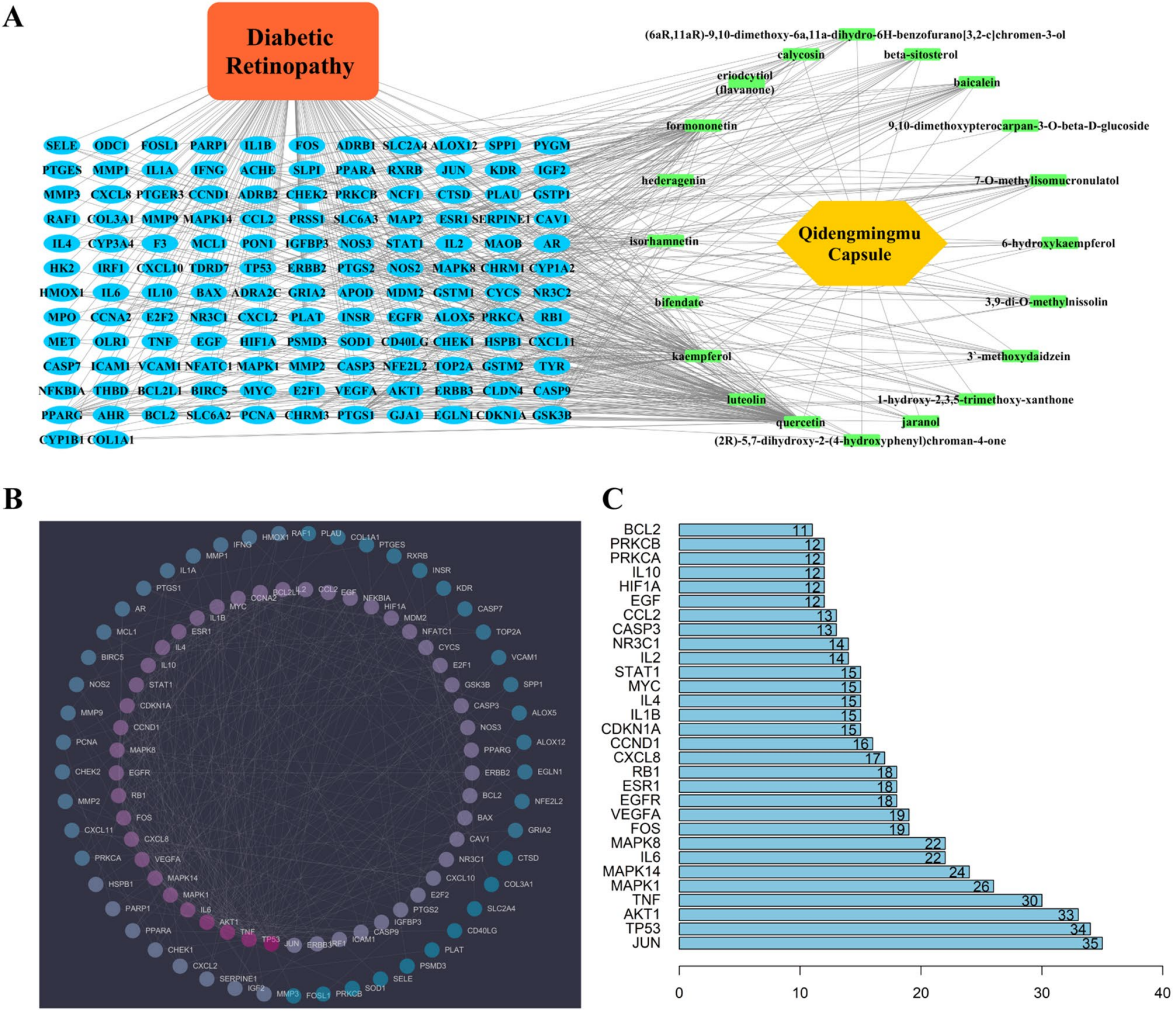
Active ingredient-DR Target Network and PPI Network based on QC-DR common targets. (**A**) Active ingredient-DR target network: the active ingredients of QC (20 ingredients) are shown as green rectangular nodes, and the common targets (134 targets) are shown as blue oval nodes. (**B**) PPI network of common targets. The nodes are ordered by degree according to the color from violet (high) to blue (low). (**C**) Key proteins in the PPI network and the correlated degrees. The X-axis represents the degree of the target, and the Y-axis represents the gene symbol of the target.

We imported the active ingredients and common targets into Cytoscape 3.7.2 to construct an active ingredient-DR target network. In the network (Fig. 2A), 20 active ingredients (shown as green rectangular nodes), 134 common targets (shown as blue oval nodes) and the edges among them are clearly exhibited. The network reflects the multiple and complicated effects of active ingredients of QC on DR. The PPI information from the STRING platform was imported into Cytoscape software and used to construct a PPI network based on common targets. The protein nodes are ordered by degree according to color from deep violet (high degree) to deep blue (low degree). The median degree was five and is set as the neutral color, gray (Fig. 2B). According to the degree, the top 20 common targets are shown in a column chart (Fig. 2C). We found that several key DR-related proteins, such as AKT1, HIF1A, VEGFA and TNF α, are involved in the treatment effects of QC on DR.

### GO and KEGG enrichment analyses

**Figure 3:**
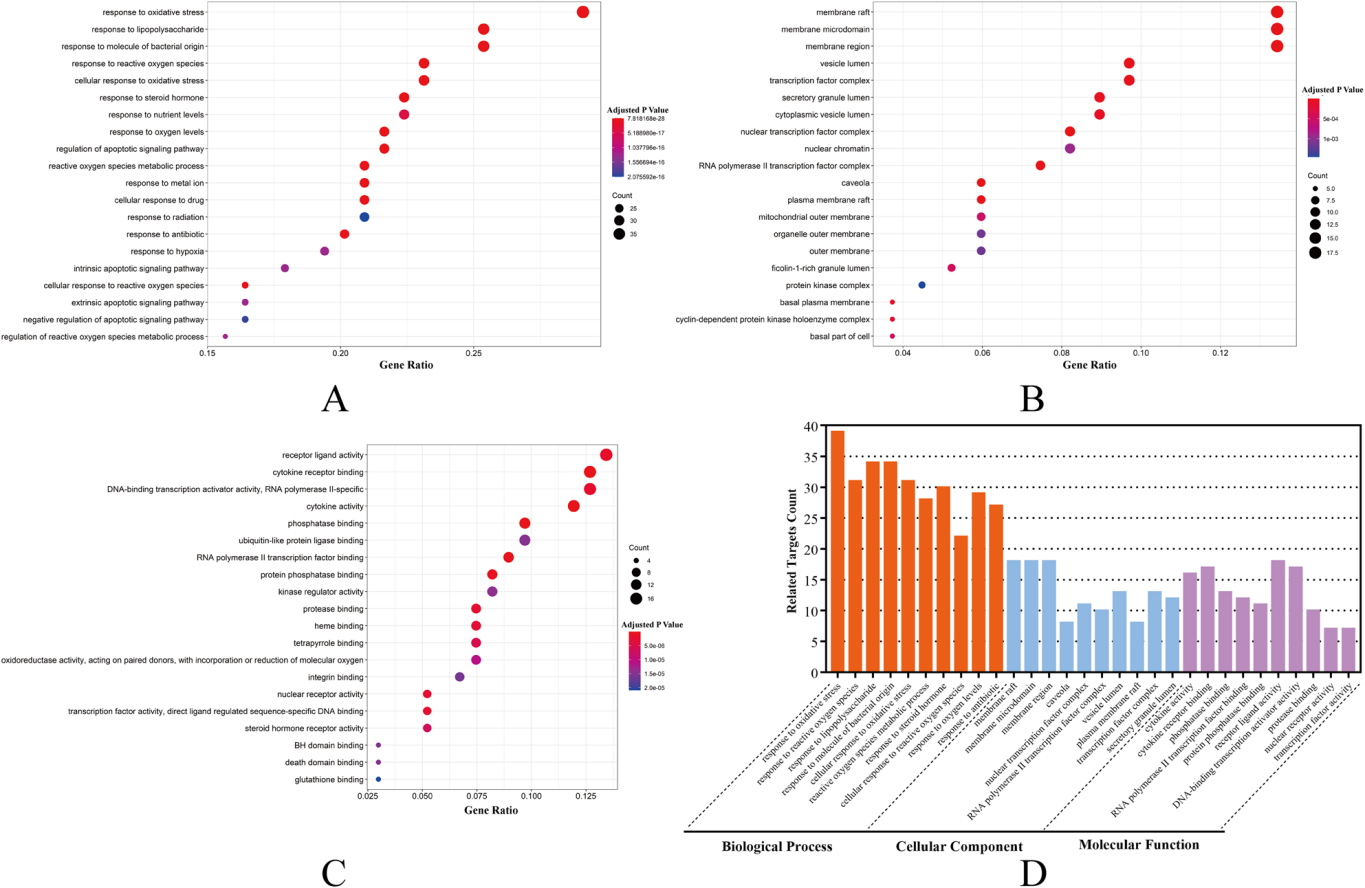
Results of GO enrichment analysis. (**A**) Bubble chart of the biological process category terms from GO enrichment analysis (the X-axis and Y-axis show the gene ratios and full names of the processes, respectively, and the color and size of each bubble represent the adjusted P value and gene count, respectively; the subsequent bubble charts are presented similarly). (**B**) Bubble chart of the cellular component category terms from GO enrichment analysis. (**C**) Bubble chart of the molecular function category terms from GO enrichment analysis. (**D**) Key processes from the results for GO categories and the counts of related target genes.

**Figure 4:**
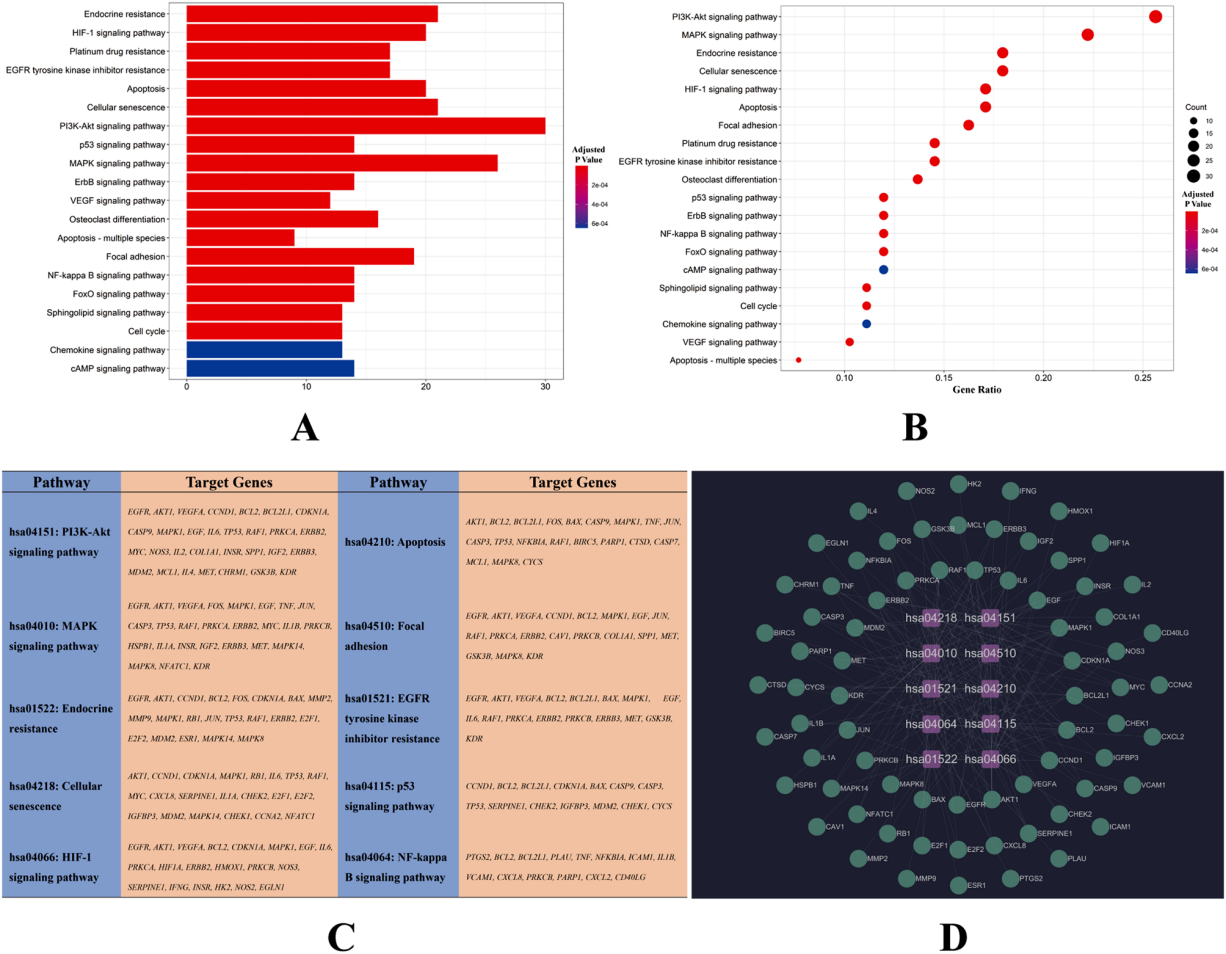
Results of KEGG enrichment analysis and key pathway network construction^19–21^. (**A**) and (**B**) Column chart and bubble chart of pathways highly relevant to the treatment effects of QC. (**C**) Top 10 signaling pathways by gene ratio and their related target genes. (**D**) KEGG key pathway network based on KEGG enrichment analysis.

Through GO enrichment analysis based on common QC-DR targets, we obtained a total of 2307 terms related to the treatment effects of QC on DR, and these terms could be divided into 3 categories (2108 terms in the biological process category, 50 terms in the cellular component category and 149 terms in the molecular function category). The top 20 terms in the 3 categories above are shown as bubble charts in Fig. 3A–C. According to the results of GO enrichment analysis, the active ingredients in QC primarily target the response to oxidative stress, the cytokine system, the activity of transcription factors and the regulation of apoptosis, which have been verified to play important roles in the incidence of DR^24,25^. The 10 biological process terms with the lowest adjusted P values in each category (a total of 30 terms) are shown in the column chart in Fig. 3D. Through the results of KEGG pathway enrichment analysis, we acquired 39 signaling pathways involved in the possible mechanism by which QC treatment affects DR and show the top 20 pathways in Fig. 4A, B according to the gene ratios. The results suggested that QC-DR common targets are concentrated mainly in VEGFA signaling, HIF-1 signaling, PI3K-Akt signaling, NF-κB signaling and apoptosis-related signaling pathways, which heavily participate in the etiology of DR. To analyze the significance and importance of key targets in the pathways involved in the treatment effects of QC, we chose 10 key pathways according to the gene ratios and adjusted P values from the results of KEGG enrichment analysis and related targets (details listed in Fig. 4C) to construct a KEGG key pathway network (Fig. 4D). Analysis of the KEGG key pathway network revealed 14 target factors involved in more than 4 pathways, which included AKT1, VEGFA, BCL2, TP53, BCL2L1, MAPK1, RAF1, PRKCA, PRKCB, ERBB2, CCND1, CDKN1A, EGF and EGFR. Based on the results of the enrichment analysis, we propose that QC could target multiple functional and biological factors in DR, but the effects and profound influence still need further research for validation.

### Key target-ingredient molecular docking analysis

**Figure 5:**
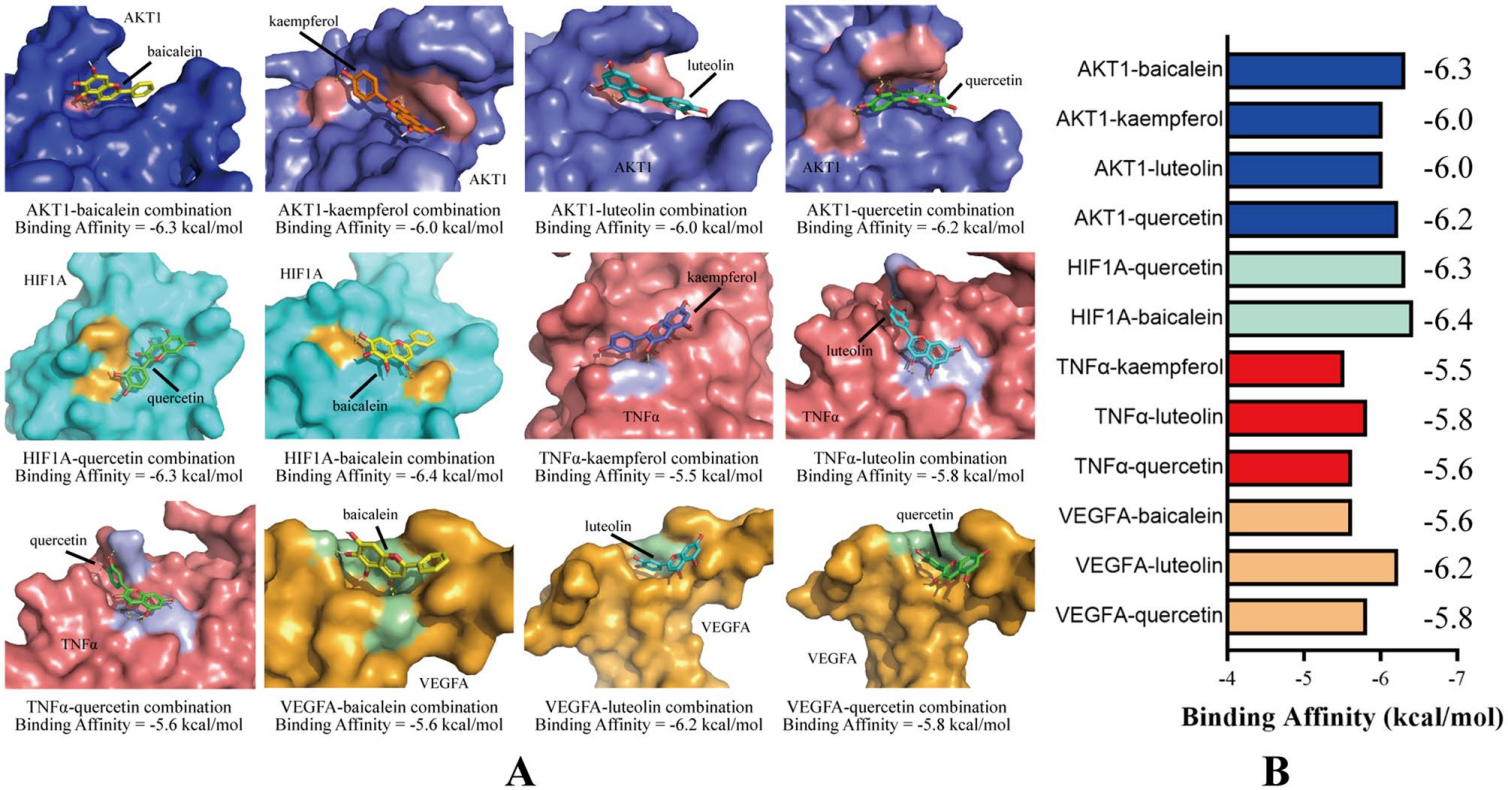
Molecular docking results. (**A**) Docking patterns of key targets (AKT1, HIF1A, TNFα, and VEGFA) and specific active ingredients (baicalein, kaempferol, quercetin, and luteolin) of herbs. (**B**) Binding affinities (kcal/mol) of key targets and specific active ingredients of herbs.

On the basis of the results of KEGG enrichment analysis, we examined several pathways that may be highly related to the incidence of DR, including the PI3K-AKT signaling pathway (which had the highest degree of gene enrichment), the HIF-1 signaling pathway (the initial pathway for retinal neovascularization), and apoptosis-related and NF-κB signaling pathways (which participate in the corruption of the blood-retinal barrier in DR)^26,27^. The PPI network suggested that AKT1, HIF1A, TNFα and VEGFA represent key targets in the pathways mentioned above, and these nodes showed importance in the PPI (with high degrees). Therefore, we selected them as DR-related key targets and selected their specific ingredients from ingredient-DR target network analysis to perform molecular docking validation. The related active ingredients included quercetin (CAS: 117-39-5), baicalein (CAS: 491-67-8), luteolin (CAS: 491-70-3) and kaempferol (CAS: 520-18-3). The crystal structures of key target proteins were obtained from the RCSB PDB; these proteins were AKT1 (1unq), HIF1A (4h6j), TNFα (5yoy) and VEGFA (4zff). It is difficult for TCM researchers to identify the accurate binding sites of target proteins for herbal ingredient molecules, and the functional binding sites of DR-related targets for ingredients of QC remain unknown. To ensure the accuracy of the docking prediction results, we used DeepSite to predict the possible docking pockets of the target proteins^23^. The docking analysis of the ingredients and proteins above was performed with the assistance of AutoDock Vina (version 1.1.2), and the binding areas were limited within the docking pockets predicted by DeepSite. The docking patterns and binding affinities are shown in Fig. 5A, B. The docking results are shown as molecular surface representations, which can effectively reflect the topical details of binding sites. The binding sites are shown in different colors on the protein surface, and hydrogen bonds are shown as dotted lines (Fig. 5A). The absolute values of binding affinities (kcal/mol) of all docking patterns were greater than 5 kcal/mol, indicating stable combination, and the details of the binding affinities are shown in Fig. 5B.

## Discussion

DR has become the leading threat to global vision health with the increasing incidence of DM and the decreasing age of DM patients. Microvascular lesions are the main pathological change in DR, and they often arise long before the occurrence of appreciable symptoms^4^. Clinical scientists have found that the foveal avascular zone (FAZ, a physiological avascular zone located in retinal macular area) in DM patients without DR is enlarged compared with that in healthy people by optical coherence tomography angiography (OCTA, a widely used non-invasive optical examination for retinal vascular structure) examination, which means that retinal degeneration in DM patients is initiated earlier than we previously thought^28,29^. QC is a traditional Chinese patent medicine (TCPM) that contains multiple active ingredients extracted from 3 herbs: *Erigeron breviscapus*, *Astragalus membranaceus* and Radix Puerariae. In in vivo experiments, QC has been proven to alleviate blood-retinal barrier (BRB, functional barrier to sustain the retinal fluid balance and to prevent leakage from vessel\choroid) dysfunction and reduce vitreous VEGF concentrations in DM model rats^30,31^. In this study, we aimed to determine the potential mechanism underlying the treatment effects of QC on DR through network pharmacology methods. The results suggested that the active ingredients of QC can target key proteins associated with multiple biological processes and signaling pathways related to DR, but the functional changes in these factors and the profound influences of treatment need to be elucidated in further animal experiments and clinical trials.

Initially, we obtained 7034 DR-related targets from the GeneCards, OMIM and GEO databases. The TCMSP database was used to acquire 28 active ingredients and 193 QC-related targets from 3 herbs in QC: *Erigeron breviscapus*, *Astragalus membranaceus* and Radix Puerariae. A total of 134 QC-DR common targets and 20 corresponding active ingredients were obtained from the intersection of QC-related targets and DR-related targets with the help of R. We ranked the active ingredients of QC involved in the treatment of DR by degree in the active ingredient-DR target network in Fig. 1D, and the top 5 ingredients were quercetin (degree: 99), kaempferol (degree: 71), formononetin (degree: 58), luteolin (degree: 44) and baicalein (degree: 24). Kaempferol has been proven to protect retinal ganglion cells (RGCs, afferent neuron of visual signaling) from high-glucose environment-induced injury in vitro by promoting ERK phosphorylation and increasing vasohibin-1 (VASH1) expression^32^. Quercetin exerts protective effects on retinal pigment epithelial cells, the main functional cells in the outer BRB, in high-glucose environments by suppressing miR-29b to balance the PTEN/AKT signaling and NF-κB signaling pathways^33^. The PPI network results showed that QC-DR common targets were mainly key proteins in pathways (AKT1, VEGFA, etc.), cytokines (TNFα, IL6, etc.) and apoptosis-related factors (P53, BCL2, etc.).

The results of GO enrichment analysis demonstrated the multifaceted and complicated effects of QC and revealed several biological process terms significantly involved in the development of DR, such as the cytokine system, response to oxidative stress, activity of transcription factors and regulation of apoptosis terms. Protein kinase C (PKC), a key intracellular regulator involved in various biological behaviors, can be activated by a high-glucose environment in vascular endothelia and then constrict retinal vessels to reduce retinal perfusion^25^. In a wound scratch assay performed by Peng, a high concentration of glucose slowed healing of endothelia by stimulating Toll-like receptor 4 (TLR4) via intracellular PKC activation^34^. Li’s study demonstrated that QC effectively reduces the vitreous concentration of PKC in rats with spontaneous DM^35^. However, kinase activity is not the only impacted factor. NADPH oxidase plays an important role in the aerobic respiration of cellular mitochondria, and our results showed that NADPH is one of the target proteins of QC. Hyperglycemia-induced TLR4 activation can influence the activity of NADPH in mitochondria, promote the production of reactive oxygen species and ultimately result in apoptosis by disrupting the balance between autophagy and apoptosis^36^. Oxidative stress has been proven to play an important role in diabetes-related vascular damage^37^. Elevated intracellular glucose concentrations can promote accumulation of sorbitol, and excess sorbitol results in microvascular dysfunction caused by induced endothelial cell apoptosis, basement membrane thickening and pericyte injury^38^. Elmazoglu’s experiment proved that luteolin can effectively alleviate microglia-induced neuroinflammation by altering oxidative stress reactions^39^. Baicalein exerts therapeutic effects on diabetic complications by inhibiting oxidative stress and inflammatory reactions via PI3K-Akt signaling^40^.

**Figure 6:**
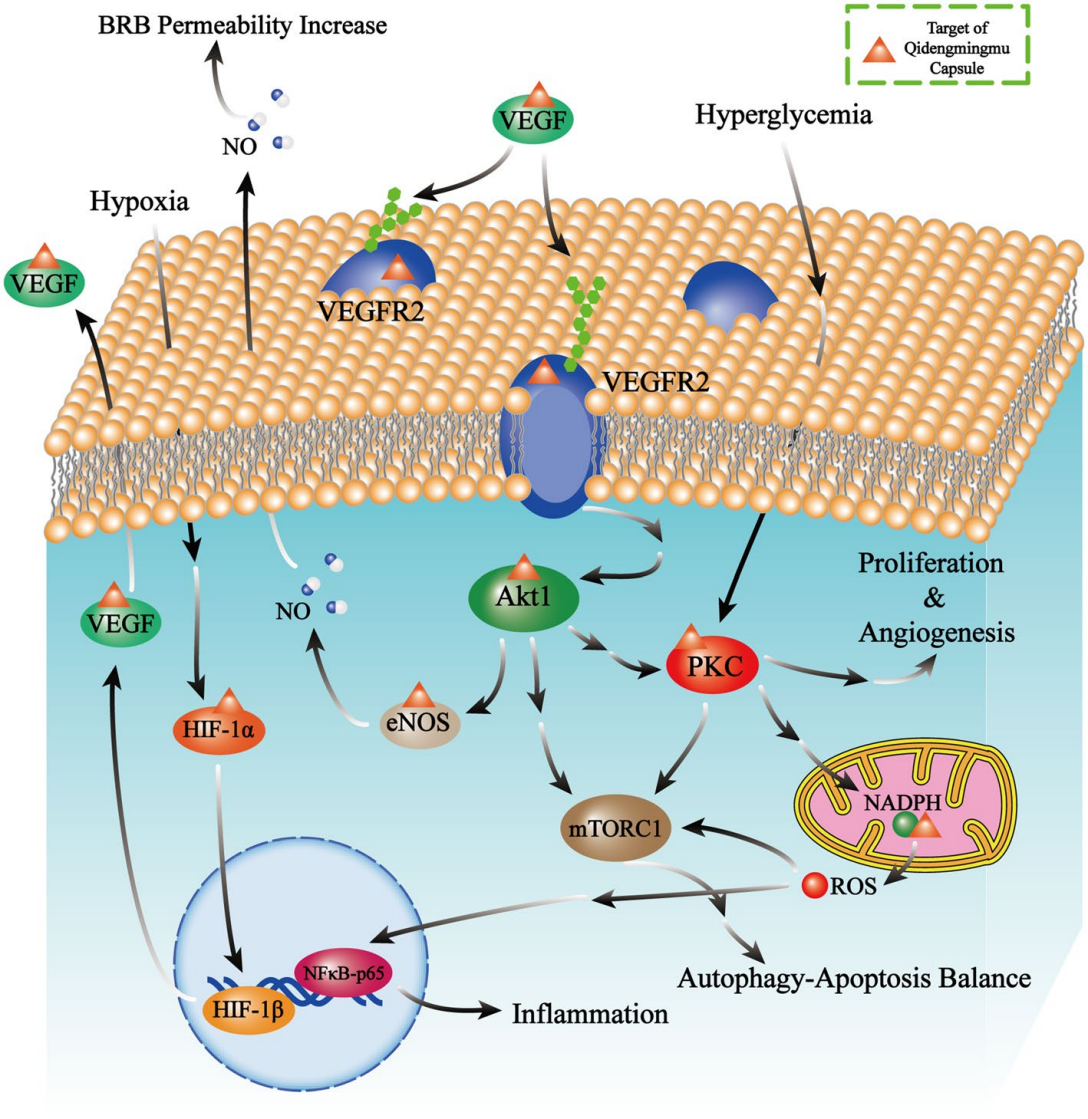
Schematic diagram of important pathways and key targets of QC in the treatment of DR. The proteins labeled with small orange triangles are target proteins of QC, the single arrows represent the direct actions, and the double arrows represent indirect actions. Some intermediate pathway-related molecules are omitted.

The results of our KEGG enrichment analysis revealed many signaling pathways that are involved in the development and progression of DR, including the PI3K-Akt, HIF-1, VEGF, and NF-κB signaling pathways. Activation of the HIF-1 signaling pathway in tissues and cells is considered an adaptive behavior to a hypoxic environment. Active HIF-1α, the key protein in HIF-1 signaling, can induce metabolic reactions, neovascularization, survival, proliferation, and conditional apoptosis^41^. Inhibition of HIF-1α in DR can reduce the expression of VEGF and prohibit neovascularization in vivo^26^. Subsequent activation of PKC and AKT1 can be induced by VEGF signaling and then promote endothelial proliferation and increase neovascular permeability, which are highly related to retinal exudation and macular edema in DR^27, 42^. The permeability change of the BRB is partly attributable to increased phosphorylation of endothelial nitric oxide synthase (eNOS), which directly mediates the synthesis and release of nitric oxide (NO), and NO is an important regulator in vasodilatation that can increase microvascular permeability^43,44^. The balance between autophagy and apoptosis plays an important role in neovascularization in DR, and in vitro experiments have proven that autophagy inhibitors effectively reduce retinal vascular endothelial migration and tube formation^45^. As the common downstream molecule of PI3K/Akt signaling and the PKC pathway, mTORC1 can mediate autophagy and apoptosis balance^45,46^. The PI3K/Akt signaling pathway is closely related to the cell cycle, and its key protein AKT1 has multiple interactions with members of the P53 signaling pathway. P53, a transcription factor, is a regulator of apoptosis-related proteins, and it participates in the disruption of the BRB in DR^47^. The NF-κB signaling pathway regulates the expression of multiple inflammatory factors, and suppressing the activity of NF-κB signaling in the retina can effectively attenuate DR-related damage^48,49^. We identified key proteins targeted by active ingredients of QC in the pathways above and show the related biomechanism in Fig. 6. The KEGG and GO enrichment analysis results provide us with a potential mechanistic explanation for the treatment effects of QC, but further animal experiments and clinical trials are still needed to prove the rationality of QC treatment. A previous study performed by Li explored certain treatment effects of Ge-Gen-Qin-Lian Compounds on type 2 diabetes mellitus and provided systematic biological evidence of formula synergistic effects through network topology, clustering analysis and GO enrichment analysis. Furthermore, Li and his colleagues verified that 4-hydroxyphenytoin (a key ingredient of Ge-Gen-Qin-Lian Compounds) exerts antidiabetic effects by stimulating insulin secretion and improving insulin resistance^50^.

In addition, we performed molecular docking on screened active ingredients and key targets to validate the results of network pharmacology. Based on the PPI network and KEGG enrichment analysis, we chose 4 key target proteins closely related to the development of DR: AKT1, HIF-1Α, TNFα and VEGFA. The active ingredients that target these proteins, including quercetin, baicalein, kaempferol and luteolin, were acquired from TCMSP. We obtained the docking results and visualized the docking within the docking pockets predicted by DeepSite using AutoDock Vina and PyMOL software, and the results are shown in Fig. 5A. The binding affinities of the docking results ranged from -5.5 to -6.4 kcal/mol, which indicated stable binding. HIF1A had the least average binding affinity among the four target proteins we selected at -6.4 kcal/mol. As it is a virtual screening method, molecular docking involves unpredictable bias from reality, which could lead to some errors in experiments in vivo/in vitro. However, the docking results reflect possible treatment mechanisms and provide guidance for animal validation experiments. Presently, network pharmacology is widely utilized for TCM mechanism research due to its powerful ability to analyze complicated relationships among multiple ingredients and numerous targets. In addition, network pharmacology effectively elucidates the combinatorial effects of TCMs based on biomedical and systematic biology methods and accelerates the TCM modernization process. Docking technology provides an efficient method to estimate the binding modes of herbal ingredient molecules with disease-related key target proteins, which can also help researchers choose potential herbal ingredients on which to perform experiments in vivo or in vitro^9^. However, multiple ingredients in a TCM formula may target the same protein and may cause synergistic or antagonistic effects during therapy, which could be neglected by the current one ligand-one protein docking modes^51^.

## Conclusion

In this study, we found that the potential mechanisms of treatment are closely related to processes such as neovascularization, autophagy, apoptosis, and inflammation, and these biological behaviors are regulated by the PI3K/Akt, VEGFA, and NF-κB pathways, among others. The molecular docking results suggest that the active ingredients of QC stably bind with key proteins, namely, AKT1, HIF-1Α, VEGFA and TNFα, in the context of DR. The study has some limitations due to the lack of experimental validation, but it suggests directions for additional experiments in vitro or in vivo. Overall, our study reveals systematic biological profiles of QC. Future studies could seek to develop potential applications of QC or confirm our findings.

## Data Availability

All the data can be obtained from the open-source platform provided in the article, and conclusions can be drawn through analyses in the relevant software programs.
