# Author Correction: Network pharmacology and molecular docking study on the active ingredients of qidengmingmu capsule for the treatment of diabetic retinopathy

**DOI:** 10.1038/s41598-021-95437-1

**Published:** 2021-08-02

**Authors:** Mingxu Zhang, Jiawei Yang, Xiulan Zhao, Ying Zhao, Siquan Zhu

**Affiliations:** 1 Eye School, Chengdu University of Traditional Chinese Medicine, 37 Shi Er Qiao Road, Jinniu District, Chengdu, 610036 China; 2 National Key Laboratory of Human Factors Engineering, China Astronaut Research and Training Center, Lvyuan Road, Haidin District, Beijing, 100089 China; 3 Department of Ophthalmology, Beijing Anzhen Hospital of Capital Medical University, 2 Anzhen Road, Chaoyang District, Beijing, 100020 China

Correction to: *Scientific Reports* https://doi.org/10.1038/s41598-021-86914-8, published online 01 April 2021

The original version of this Article contained an error in Affiliation 1, which was incorrectly given as “Eye School of Chengdu, University of T.C.M., 37 Shi Er Qiao Road, Jinniu District, Chengdu, 610036, China.” The correct affiliation is listed below:

Eye School, Chengdu University of Traditional Chinese Medicine*,* 37 Shi Er Qiao Road, Jinniu District, Chengdu, 610036, China.

The original Article has been corrected.

